# The thalamus in psychosis spectrum disorder

**DOI:** 10.3389/fnins.2023.1163600

**Published:** 2023-04-13

**Authors:** Alan Anticevic, Michael M. Halassa

**Affiliations:** ^1^School of Medicine, Yale University, New Haven, CT, United States; ^2^Department of Neuroscience, Tufts University School of Medicine, Boston, MA, United States

**Keywords:** thalamus, psychosis, cognition, functional connectivity, pharmacology, mediodorsal nucleus, computation

## Abstract

Psychosis spectrum disorder (PSD) affects 1% of the world population and results in a lifetime of chronic disability, causing devastating personal and economic consequences. Developing new treatments for PSD remains a challenge, particularly those that target its core cognitive deficits. A key barrier to progress is the tenuous link between the basic neurobiological understanding of PSD and its clinical phenomenology. In this perspective, we focus on a key opportunity that combines innovations in non-invasive human neuroimaging with basic insights into thalamic regulation of functional cortical connectivity. The thalamus is an evolutionary conserved region that forms forebrain-wide functional loops critical for the transmission of external inputs as well as the construction and update of internal models. We discuss our perspective across four lines of evidence: First, we articulate how PSD symptomatology may arise from a faulty network organization at the macroscopic circuit level with the thalamus playing a central coordinating role. Second, we discuss how recent animal work has mechanistically clarified the properties of thalamic circuits relevant to regulating cortical dynamics and cognitive function more generally. Third, we present human neuroimaging evidence in support of thalamic alterations in PSD, and propose that a similar “thalamocortical dysconnectivity” seen in pharmacological imaging (under ketamine, LSD and THC) in healthy individuals may link this circuit phenotype to the common set of symptoms in idiopathic and drug-induced psychosis. Lastly, we synthesize animal and human work, and lay out a translational path for biomarker and therapeutic development.

## Introduction

Psychosis spectrum disorder (PSD) is a pervasive and debilitating syndrome that affects tens of millions of people worldwide, with a lifetime risk of about 1% regardless of geography (Rehm and Shield, 2019). Patients with PSD experience symptoms that greatly impact their daily functioning (Picchioni and Murray, 2007; Rehm and Shield, 2019), including hallucinations, delusions, disorganized speech, loss of motivation, flat affect, as well as pervasive deficits in cognitive control (Barch and Ceaser, 2012). Cognitive control deficits are a major focus for research and treatment development, as they are relatively unaffected by current medications and contribute to overall morbidity and loss of workforce by the illness (Auquier et al., 2006; Kooyman et al., 2007). Critically, PSD patients vary greatly in their symptom expression and consequently, appropriate stratification and connection to neurobiological mechanisms has been challenging (Jablensky, 2010; Foss-Feig et al., 2017; Ji et al., 2021). Nonetheless, advances in both human and non-human animal work over the last decade may provide mechanistic targets for PSD symptoms. For example, it is now widely recognized that PSD is associated with *functional dysconnectivity* across distributed neural systems measured using non-invasive human neuroimaging (Woodward et al., 2012; Anticevic et al., 2014a,b, 2015; Woodward and Heckers, 2016; Giraldo-Chica and Woodward, 2017; Murray and Anticevic, 2017; Baran et al., 2019; Sheffield et al., 2020; Avram et al., 2021; Fryer et al., 2021; Huang et al., 2021; Ji et al., 2021, 2019a; Kraguljac and Lahti, 2021; Kraguljac et al., 2021; Abram et al., 2022; Li et al., 2022; Sabaroedin et al., 2022). Identifying the circuit mechanisms underlying the functional systems-level alterations observed with neuroimaging is critical to rationally guide therapeutics for specific symptoms at the level of individual patients, especially those that do not respond adequately to antipsychotics. To make this goal possible, it is critical to focus on translationally tractable neural targets linked to specific symptoms, which can be effectively studied in animals and humans.

**FIGURE 1:**
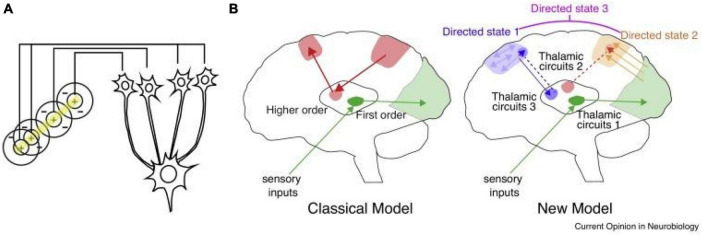
Thalamic control of functional cortical connectivity. **(A)** The classical transformation of visual representation from center/surround to orientation tuning involves drive from the lateral geniculate nucleus (LGN) to primary visual cortex, where the resulting cortical representation is dependent on thalamic input (information transmission). **(B)** The classical model reflects the idea of information relay from the LGN, which is shared by other sensory systems (green), and extended to account for thalamic nuclei that receive driving inputs from the cortex rather than subcortical or sensory inputs. The new model suggests that many of these circuits operate to enhance connectivity within a cortical region (e.g., blue), or across different cortical areas (e.g., orange). In these scenarios thalamic inputs do not necessarily drive spiking (do not dictate cortical spike times), but rather enhance how spike times are generated in response to specific inputs (either local, or from area A, B, C, etc.). A particular connectivity pattern reflects a directed arousal state, as defined in this review. Such states can encompass multiple cortical nodes and utilize multiple thalamic amplifiers (e.g., directed state 3), depending on the complexity of the associated cognitive process.

Like all mammalian counterparts, the human forebrain is organized into loops connecting the cortex, thalamus and basal ganglia (Shepherd and Yamawaki, 2021). Broadly, the cortex is largely composed of recurrent excitatory circuits that are capable of maintaining activity patterns across multiple time scales relevant to driving adaptive behavior (Macpherson et al., 2021). Local cortical interneurons gate and sculpt these activity patterns (Averbeck and O’Doherty, 2022). The basal ganglia are inhibitory structures that are largely driven by cortical inputs, and gated by reward-related signals (e.g., dopamine), allowing them to function at least in part as critical nodes in reinforcement learning (Averbeck and O’Doherty, 2022). The thalamus is a centrally located subcortical region that is divided up into multiple nuclei, some of which receive basal ganglia input, and almost all of which have extensive, segregated and well-organized connections to the cortex (Halassa, 2011; Nakajima and Halassa, 2017; Rikhye et al., 2018b). Because of the intimate relationship between the cortex and thalamus and the functional loops they form, they are often collectively referred to as the “thalamocortical system.” Importantly, the observed neuroimaging functional dysconnectivity in schizophrenia (and PSD more broadly) is likely a feature of a perturbed thalamocortical system (Anticevic et al., 2014a; Murray and Anticevic, 2017). This point is critical, because an increasing number of studies indicate that the thalamus may be a particularly attractive region to target in a number of brain disorders (Lee et al., 2019). The key for each disorder may be in the type of connectivity patterns the targeted thalamic subregion establishes with the cortex. The central thalamus, for example, has widespread connections to all cortical areas and can be targeted with deep brain stimulation to reverse anesthesia in animals (Bastos et al., 2021) and restore arousal in human disorders of consciousness (Schiff et al., 2007). The anterior thalamus, which has connections to medial temporal lobe structures, can be targeted to effectively control seizures in medication-resistant epilepsy (Ryvlin et al., 2021). Therefore, the thalamus can be perhaps viewed as a spatially restricted access point to large-scale cortical networks, but with each thalamic region providing a unique opportunity for functional correction (Figure 1).

**FIGURE 2:**
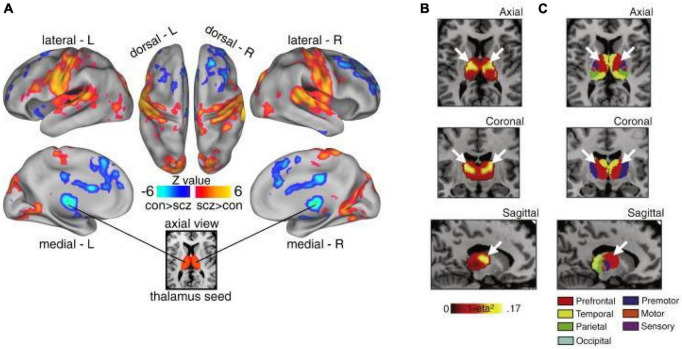
Thalamic dysconnectivity in SCZ. **(A)** Significant whole-brain between-group differences in thalamic connectivity between healthy controls (CON) and individuals with schizophrenia (SCZ). Red-orange (blue) foci mark areas where patients exhibited stronger (reduced) thalamic coupling. **(B)** Intrinsic thalamic dysconnectivity pattern based on group dissimilarity. The brightest voxels are associated with highest between-group differences. **(C)** Thalamus subdivisions based on the FSL thalamic atlas, defined by their connectivity to different regions of cortex. White arrows indicate the correspondence between thalamic regions with greatest dysconnectivity in SCZ and thalamic subdivisions strongly connected with prefrontal cortex (red). Adapted from Anticevic et al. (2014a).

In addition to its unique anatomical features, converging lines of evidence suggest that the thalamus is prominently altered in patients with PSD and schizophrenia in particular, thus supporting the idea that it may not only be a target of intervention, but also a viable biomarker of specific symptoms associated with PSD (Behrendt, 2006; Cronenwett and Csernansky, 2010; Steullet, 2020). Interestingly, numerous studies have repeatedly shown altered thalamic structure and function, even before the onset of symptoms, suggesting a role in etiology (Andreasen, 1997; Ferrarelli and Tononi, 2011; Woodward et al., 2012; Anticevic et al., 2014a,^2015^; Pergola et al., 2015; Ji et al., 2019a; Ramsay, 2019; Steullet, 2020). Of note, some studies have shown that higher-order, associative thalamic nuclei may be particularly affected in schizophrenia relative to primary sensory nuclei (Andreasen, 1997; Anticevic et al., 2014a; Dorph-Petersen and Lewis, 2017; Sherman, 2017; Steullet, 2020) (Figure 2). This may reflect a fundamental difference in the vulnerability of associative thalamocortical systems to disease mechanisms that ultimately produce PSD symptoms. Specifically, in contrast to primary sensorimotor systems, prefrontal and associative cortical areas handle a variety of input types that require dynamic gating based on changing cognitive task demands and behavioral goals (Hazy et al., 2007). This may place associative thalamocortical systems in a particularly vulnerable state by disease processes that affect basic synaptic transmission and plasticity, both with genetic substrates known to be altered in schizophrenia (Anticevic and Lisman, 2017). Consistent with this notion is the clinical literature showing that localized vascular lesions in associative thalamic structures can actually lead to psychosis, particularly when it impacts projections to prefrontal cortical regions (Crail-Melendez et al., 2013).

Here we discuss a framework that considers the critical role of the thalamus in the neurobiology of PSD. We structure this piece into four sections. First, we outline our central hypothesis related to thalamocortical dysconnectivity and PSD symptomatology, with an overarching framing that PSD fundamentally affects computations supporting cognitive control and reasoning (Garety et al., 2005; Cardella and Gangemi, 2015; Burgher et al., 2021). Second, we discuss recent animal studies that have redefined the role of the thalamus in cognitive function, laying the groundwork for how its dysfunction can contribute to cognitive control and reasoning deficits in PSD. Third, we present robust human neuroimaging evidence that strongly implicates thalamic functional alterations in PSD, which opens a promising domain for treatment-relevant biomarker development in relation to specific PSD symptoms. Here we place an emphasis on studies that use so-called resting-state functional connectivity (rs-FC), which offers a powerful non-invasive tool for quantifying system-level thalamocortical coordinated activity at the single patient level. We link these findings to an emerging pharmacological imaging literature in healthy individuals, which shows how transient administration of specific drugs (e.g., ketamine, LSD, or THC) can mimic not only certain behavioral symptoms of PSD, but also key aspects of the rs-FC “thalamocortical dysconnectivity” observed in PSD patients. Fourth, we synthesize the animal and human work into a translational framework focused around thalamocortical systems in PSD, and speculate on a rational path for biomarker and therapeutic development.

## Psychosis spectrum disorder as a dysconnectivity syndrome

A major idea in this piece is that PSD is a syndrome associated with perturbation in processing across distributed large-scale neural systems, which preferentially and severely impacts forebrain associative systems. This *functional dysconnectivity* underlies the debilitating, complex and highly heterogeneous symptoms seen in PSD patients. These symptoms include formal thought disorder, delusional beliefs, hallucinations, affective disturbances, and executive deficits (e.g., short-term memory, task switching, planning, logical reasoning to name a few). We suggest that all of these symptoms are to some extent cognitive in nature, and are linked to perturbations in coordinated activity patterns across higher-order associative circuits. Within this framework, associative thalamic circuits play an essential regulatory role, offering a window into PSD etiology and perhaps even rational correction.

It is important to recognize that the clinical reality of PSD is heterogeneity; the symptoms outlined above are variable in onset, severity, prevalence, and treatment response across individual patients. Therefore, it is likely impossible to define this syndrome by a single neurobiological “cause.” Instead, we suggest that the core deficits across the spectrum of psychotic illness are related to appropriately organizing information flow across levels of reasoning hierarchy (Hilgetag and Goulas, 2020). In other words, appropriately processing, maintaining, and inferring the causes of events in highly complex dynamic environments, particularly when many of them are unobservable, is perturbed in schizophrenia. There is now ample evidence to suggest that such operations are serial in nature, and require the iterative interactions between multiple frontal systems, each of which specialized for a particular class of operations within any given cognitive algorithm (e.g., orbitofrontal cortex in constructing value estimates) (Padoa-Schioppa and Conen, 2017), dorsolateral prefrontal cortex in coordinating and updating of task sets (Soltani and Koechlin, 2022), anterior cingulate in generating confidence weighted error signals (Sarafyazd and Jazayeri, 2019). The coordinated computations across these regions is effectively the neural substrate of forming internal representations upon which reasoning models are built (Langdon et al., 2022).

The key aspect of the framework that we offer here, is that the associative thalamus provides a functional bridge across these specialized frontal areas, allowing their interactions to be both dynamically efficient and highly flexible, depending on the behavioral context (Schmitt et al., 2017; Rikhye et al., 2018b; Hummos et al., 2022). Therefore, perturbations within any of these core associative cortical areas, the thalamus itself, or their interactive computations is likely to result in a variety of cognitive disruptions at the behavioral level, which present in patients along the psychosis spectrum. Perhaps more critically, is an essential neurobiological property whereby the thalamus represents a bottleneck for information exchange between distributed association cortical areas. Therefore, we posit that the thalamus may be a particularly sensitive access point for intervention in ways that may remain agnostic to where the primary perturbation actually is.

## The functional role of thalamus in cognition

As noted, the thalamus is a centrally-located structure in the mammalian forebrain, which is traditionally divided into individual nuclei based on Nissl staining and gross connectivity (Jones, 2007). There are over fifty distinct nuclei, which may be grouped into seven major anatomical divisions: anterior, medial, lateral, ventral, intralaminar, midline and posterior (Rikhye et al., 2018b). At the microcircuit level, these nuclei are largely composed of excitatory projection neurons that are devoid of local lateral connections (Jones, 2007; Rikhye et al., 2018b; Halassa and Sherman, 2019). The thalamic reticular nucleus is a shell of GABAergic neurons that surrounds these excitatory nuclei (Pinault, 2004; Crabtree, 2018; Takata, 2020). In primates, excitatory thalamic nuclei contain local inhibitory interneurons (Jager et al., 2021), but little is known about their function beyond a few well-studied cases. For example, in the lateral geniculate nucleus, interneurons are thought to engage in a push-pull type of operation that ultimately improves signal-to-noise ratio of thalamocortical visual input transmission (Hirsch et al., 2015). Because much basic physiology is carried out in rodents, and the rodent thalamus is devoid of local interneurons outside of the lateral geniculate, we know little about the general properties of these circuits. Instead, considerable focus has been placed on the reticular nucleus, which provides a major source of inhibition to thalamic projection neurons across all nuclei (Halassa et al., 2014; Wimmer et al., 2015; Halassa and Acsády, 2016).

Given the lack of local excitatory recurrence in the thalamic nuclei, the functionality of thalamic projection neurons is anchored in their long-range input-output connectivity patterns (Halassa and Sherman, 2019). Arguably, the best understood thalamic structures are those involved in sensory processing, such as the lateral and medial geniculate (vision and audition), and the posteromedial nucleus (somatosensation). These structures are often referred to as thalamic “relays,” transmitting inputs from sensory organs to cortex, with minimal spatial transformation of these sensory signals en route to their primary cortical targets. The idea is consistent with the observation that lateral geniculate neurons, which are arguably the best studied in the entire mammalian thalamus, show spatial responses very similar to those exhibited by their retinal inputs (Hubel, 1960). In fact, individual geniculate neurons are driven by as few as three retinal ganglion cell inputs with similar on-off spatial responses (Usrey et al., 1999), suggesting that the thalamus is a computationally-redundant station from the retina to primary visual cortex [although this argument is challenged in the temporal domain (Briggs and Usrey, 2005)]. In this pathway, the transformation from center-surround seen in retina to oriented edges and local motion vectors happens at the thalamo-recipient cortical layer (Layer IV) (Reid and Alonso, 1995).

Recent studies outside of primary sensory thalamic areas have challenged the relay view, and ascribed a critical functional role for the thalamus in cognitive computations. For instance, studies in associative thalamic structures like the mediodorsal thalamus and pulvinar have shown that these nuclei may have convergent inputs from multiple cortical areas and that, in contrast to primary relay nuclei, they can integrate and transform these inputs into novel representations, including temporal context (Rikhye et al., 2018a), confidence (Komura et al., 2013) and sensory uncertainty (Mukherjee et al., 2021). Such representations, in turn, may be utilized as cognitive control signals that functionally regulate distributed cortical dynamics rather than relay signals in the classical sense (Halassa and Kastner, 2017).

Consistent with this revised model of the thalamus (Figure 1), work in the non-human primate pulvinar has shown that in attentional tasks, this thalamic structure plays critical roles in coordinating activity patterns across extrastriate cortical areas (Halassa and Kastner, 2017). Specifically, work by Saalmann et al. (2012) has shown that interactions between early and late stations of the dorsal visual pathway in the alpha-band range, are temporally coordinated by an intermediary connecting pulvinar. More recently, the same group showed that this relationship is causal; inactivation of the pulvinar diminishes this cortico-cortical alpha band coherence in a task-specific manner (Eradath et al., 2021). Work by Komura et al. (2013), also in the non-human primate, has shown that pulvinar neurons generate summary statistic type responses in a classical visual dot motion task. Specifically, when animals are required to make a perceptual judgment on these well-studied stimuli, pulvinar neurons show response patterns that are not reflective of the dominant motion direction, as is the case in cortical area LIP (lateral intraparietal) for example, but rather their responses reflect the coherence as a scalar quantity regardless of motion direction. Computational modeling of these responses suggests they may emerge due to convergence of these two motion-sensitive cortical ensembles onto single pulvinar neurons (Jaramillo et al., 2019). Therefore, responses of primate pulvinar neurons are not a simple reflection of upstream inputs, but under certain conditions can reflect a transformation of a vector input to a scalar signal. Adding Kastner’s work to these findings paints a picture in which the pulvinar may not “relay” such signals, but rather generate an output that coordinates activity patterns across distributed cortical ensembles (Halassa and Kastner, 2017).

Similar to the pulvinar, the mediodorsal thalamus establishes widespread connections across multiple cortical areas (Phillips et al., 2021). However, rather than being connected to higher-order visual areas, the mediodorsal thalamus is connected to multiple frontal cortical areas (Giguere and Goldman-Rakic, 1988). Recently, multiple studies have examined mediodorsal function in rodents, and showed that neurons within this region have non-relay properties that might be similar to the pulvinar studies described above. Specifically, in a cross-modal divided attention task developed in mice, mediodorsal neurons show unique response profiles that are distinct from their connected cortical areas. In the prefrontal cortex, neurons encode the inputs and outputs of the cross-modal task, whereas thalamic neurons encode changes in input patterns over both long and short timescales. Encoding of the long term input changes reflects the statistical regularity of inputs over multi-trial timescale, or the “cueing context” (Rikhye et al., 2018a). Encoding of short term input changes reflects their variance or uncertainty (Mukherjee et al., 2021). This may reflect an interesting point of divergence between the mediodorsal encoding of uncertainty versus the pulvinar encoding the opposite, confidence (Komura et al., 2013; Mukherjee et al., 2021). Critically, these uncertainty responses have not only been found in non-human animal studies, but also in task-based fMRI in humans (Grinband et al., 2006; Kosciessa et al., 2021). Therefore, although thalamic microcircuitry may be far simpler than that of the cortex, it is nonetheless likely capable of performing important computational transformations that are critical for coordination of distributed cortical signals and in turn cognitive operations. The rodent studies have also shown that the thalamic outputs do not necessarily drive cortical responses in the classical sense, but can drive feedforward inhibition to slow cortical dynamics in certain contexts, or alternatively disinhibit cortical ensembles to implement non-linear gain control of cortical activity patterns in others (Delevich et al., 2015; Logiaco et al., 2021).

In summary, the fact that associative thalamic structures provide a mechanism for regulating associative cortical computations in a precise manner, and in turn coordinate activity across large-scale cortical networks is consistent with our overall thesis that associative thalamic structures could be leveraged to identify and target cognitive control deficits in PSD.

## Evidence for thalamocortical circuit alterations in psychosis spectrum disorders

### BOLD neuroimaging

Advancing research in the field of PSD will require innovation in non-invasive neuroimaging which will provide the opportunity to link observed neural changes to the diversity of symptoms in this syndrome. More specifically, progress will be accelerated by neuroimaging signals that can be optimized for risk prediction, diagnostics, treatment development and prognosis. Here we argue that the thalamus presents a uniquely powerful opportunity for PSD neuroimaging biomarker development which may be linked to specific symptoms.

Human neuroimaging has witnessed nothing short of a revolution since the discovery of a phenomenon called “resting-state,” which relies on the coordinated spontaneous activity of large-scale neural systems that can be imaged *via* functional magnetic resonance imaging sensitive to blood-oxygen-level-dependent (BOLD) signal changes. It is now widely accepted that BOLD fMRI signal exhibits spatiotemporal coherence in humans. These relationships can be quantified using statistical association (i.e., Spearman’s correlation) (Biswal et al., 1995, 2010; Fox and Raichle, 2007; He et al., 2009; Deco et al., 2011), which in turn reveal large-scale spatio-temporal coordination between neural systems; i.e., resting-state functional connectivity (rs-FC). For instance, a canonical system that can be reliably quantified *via* rs-FC is the “default mode network”–a set of cortical areas that typically show strong suppression during active cognitive task performance (Shulman et al., 1997; Mantini et al., 2011; Anticevic et al., 2012a; Raichle, 2015). This approach can be extended, in principle, across any cortical area (Biswal et al., 1995; Yang et al., 2007; Zhou et al., 2007; Andoh et al., 2015; Taren et al., 2017) or subcortical structure (Cao et al., 2009; Roy et al., 2009; Tang et al., 2011; Szewczyk-Krolikowski et al., 2014; Schleifer et al., 2019), with increasing quantitative sophistication (Allen et al., 2011; Suk et al., 2016; Haak et al., 2018; Shafiei et al., 2020; Ji et al., 2021). Therefore, the “resting state” modality provides a very attractive approach to characterize disruptions in wide-spread neural systems that may occur in neuropsychiatric illness.

Indeed, studies in PSD and schizophrenia specifically have been some of the first clinical applications of rs-FC approaches to test the prevailing hypothesis that this illness causes disruptions in large-scale functional neural connectivity. Here the thalamus presented an ideal neural target for characterizing rs-FC alterations in schizophrenia (Anticevic et al., 2014a; Wang et al., 2015; Giraldo-Chica and Woodward, 2017). Indeed, the thalamus is a region which has emerged as an important locus of dysfunction across PSD neuroimaging studies, building on years of theory implicating its role in psychosis (Andreasen, 1997; Pergola et al., 2015; Steullet, 2020) (Figure 2). Examining functional and structural thalamic macro-connectivity capitalizes on key properties of this subcortical structure: (i) as noted, it is topographically connected to the entire cortex across its many nuclei, and thus may represent a node particularly sensitive to brain-wide neural disturbances that may cause symptoms observed in schizophrenia; (ii) the thalamus contains segregated nuclei, providing a lens for examining parallel yet distributed large-scale structural and functional dysconnectivity in schizophrenia patients, thus bypassing an otherwise daunting feature mapping problem; (iii) identifying thalamic nuclei can be reliably automated on an individual subject basis *via* whole-brain neuroimaging segmentation tools that leverage high-resolution structural scans (Fischl et al., 2004, 2002), which in turn allows for reliable and robust estimation of its functional coupling and/or task-evoked signals.

The first evidence of disrupted thalamo-cortical rs-FC in schizophrenia was reported by Welsh et al. (2008) in a small sample of *n* = 11 patients. They found that the mediodorsal thalamus exhibited rs-FC reductions with the prefrontal cortex in this initial study. This preliminary but promising effect was subsequently confirmed by Woodward et al. (2012) in a much larger sample of chronic schizophrenia patients. Specifically, they “seeded” large cortical areas that are known to form distinct patterns of cortico-thalamic connectivity and tested if these cortical territories form distinct patterns of rs-FC onto the thalamus in patients relative to healthy controls (Woodward et al., 2012). First, they replicated the Welsch effect showing reduced connectivity from the prefrontal cortex onto the mediodorsal thalamus. Second, they also discovered that sensorimotor cortices exhibit increased thalamic connectivity onto thalamic nuclei in patients with schizophrenia. This phenomenon of bi-directional disruption gives rise to the notion that there is indeed a lack of coordinated information flow through the thalamus in PSD, but in a way that uniquely affects association cortex relative to primary cortical areas (Woodward et al., 2012). A parallel study (Anticevic et al., 2014a) showed that when “seeding” the thalamus specifically (as opposed to large cortical regions), patients with schizophrenia exhibit a robust, bi-directional shift in thalamocortical connectivity across large-scale cortical areas. More specifically, this study found that associative thalamic nuclei show elevated rs-FC with sensorimotor cortical areas but at the same time reductions in rs-FC with prefrontal cortex, dorsal striatum and cerebellum – again supporting the notion of disrupted cortico-thalamic information flow. In addition, Anticevic et al. (2014a) showed that this effect extends across the PSD as it was also apparent (albeit to a lesser extent) in bipolar patients with psychosis history. This core observation of thalamic hypo/hyper connectivity at rest has been replicated a number of times to date in chronic patients with schizophrenia (Anticevic et al., 2015; Woodward and Heckers, 2016; Avram et al., 2018; Wu et al., 2022). It is not an overstatement at this point to say that this is one of the more robust effects in psychiatric neuroimaging literature; for instance, this finding was replicated in a large meta-analysis conducted by Ramsay (2019) that combined 20 experiments from 17 publications that explored resting-state alterations in schizophrenia.

This robust and highly characteristic clinical neuroimaging observation could be leveraged to characterize PSD symptom heterogeneity. There are two fundamental ways in which patients with schizophrenia vary: First, individual patients vary in terms of symptom type and severity; Second, patients vary in terms of specific illness stage (e.g., prodrome versus early course versus chronic syndrome) (Kay et al., 1986; Schultz et al., 2007; Häfner et al., 2013). These two axes of heterogeneity likely interact; it is well understood that schizophrenia is a complex polygenic neurodevelopmental disorder (Schizophrenia Psychiatric Genome-Wide Association Study (GWAS) Consortium., 2011; Ripke et al., 2013; Purcell et al., 2014; Schizophrenia Working Group of the Psychiatric Genomics Consortium., 2014; Rees et al., 2015; Fromer et al., 2016; Germine et al., 2016; Li et al., 2017; Lam et al., 2019; Smeland et al., 2020) and that individual patients present with highly variable and distinctly complex individualized symptom progression, across prodrome, early onset, and chronic stages (McGlashan and Fenton, 1993; Parnas, 1999; MacDonald and Schulz, 2009; Cole et al., 2011). Moreover, not all patients respond equally well even to available lines of care (i.e., 1St and 2nd generation antipsychotics). This implies that there is likely not one root cause for all symptoms across the PST spectrum, or alternatively that a ‘core’ disruption may cause downstream neural alterations that in turn give rise to the broad spectrum of PSD symptoms. However, it is likely possible that no matter the cause, all symptom expression in some way corrupts information flow through the thalamus, perhaps in a symptom-specific way. In support of this notion, the pattern of thalamic under-connectivity with prefrontal cortex and over-connectivity with sensorimotor cortex has been reported in patients during early and later stages of psychosis (Woodward and Heckers, 2016), as well as individuals at high risk for psychosis (Anticevic et al., 2015). Similar to findings in idiopathic PSD, Schleifer et al. (2019) reported thalamic rs-FC alterations in youth with 22q11 deletion syndrome. This observation is vital as 22q11 deletion syndrome is a rare genetic mutation that is associated with 30× higher risk for developing psychosis later in life. While not causal, this genetic risk supports the notion that thalamic circuits may be a neural marker for psychosis risk regardless of etiology. In fact, the hyper/hypo thalamic dysconnectivity pattern was even stronger for a subset of at-risk patients who later progressed to full-blown illness, supporting the idea that its disruption may track illness severity (Anticevic et al., 2015). Collectively, these findings strongly support the model whereby thalamic dysconnectivity may be a core feature of PSD or at least a neural marker that is associated with both genetic and clinical risk, which emerges prior to full disease expression (Anticevic et al., 2015; Woodward and Heckers, 2016; Schleifer et al., 2019). This places the thalamus in a key position to develop the next generation of targeted rs-FC measures that link thalamocortical dysconnectivity to PSD symptom variation.

**FIGURE 3:**
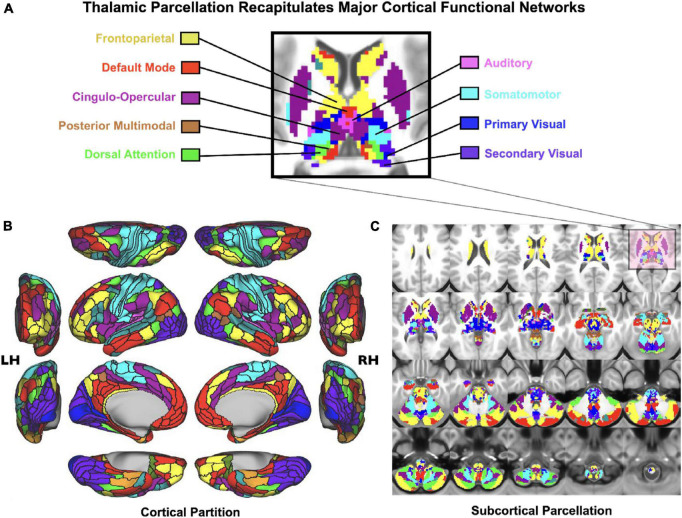
Cortico-subcortical whole-brain functional network parcellation derived from the Human Connectome Project dataset. **(A)** Zoomed in axial view onto the thalamus and striatum. Each color represents voxels mapping to a given functional network. **(B)** Cortical functional network view. **(C)** Subcortical functional network view. This effect highlights that it is possible to reliably and robustly map functional subdivisions of major sub-thalamic circuits that functionally couple with brain-wide networks Adapted from Ji et al. (2019b). This supports the notion that the thalamus indeed provides a functional “lens” onto distributed yet segregated functional pathways.

As noted, the thalamus is composed of multiple nuclei that form different types of input and output connections with cortex and striatum, in turn forming functional loops. In the context of understanding neural disruptions in PSD it is particularly important to distinguish between sensory and associative thalamus, which form distinct large-scale connection patterns onto cortex in the service of two fundamentally different types of operations: primary sensory relays from the environment versus coordination and regulation of association cortex in the service of cognitive function. Therefore, one strong prediction would be that the association thalamic connectivity may be preferentially affected by PSD – a syndrome that corrupts computations relevant for cognitive control. Consistent with this notion, the mediodorsal thalamus appears to drive the main observation of neural dysconnectivity in schizophrenia; recent studies have found reduced connectivity between higher-order thalamus and dorsal striatum, suggesting altered prefrontal cortico-striato-thalamo-cortical functional connectivity (Anticevic et al., 2014a; Avram et al., 2018; Cao et al., 2018; Ji et al., 2019a). This observation may hold the key for dissecting the roles of distinct associative thalamo-cortical loops in the heterogenous symptoms associated with PSD. The key hypothesis here is that associative thalamocortical loops would be variably affected as a function of different PSD symptoms (Peters et al., 2016). For instance, patients with thought disorder may exhibit functional alterations in thalamic circuits that connect to the lateral frontal cortex and to the fronto-parietal control network (see Figure 3). In contrast, patients with severe hallucinations (but perhaps less thought disorder) may exhibit deficits in thalamic circuits regulating sensory cortex. Of note, associative thalamic circuits like the pulvinar project to primary sensory cortical areas and regulate the balance of responsiveness to feedforward versus feedback inputs (Cortes and van Vreeswijk, 2012; Jaramillo et al., 2019). This point is critical, as the arbitration between feedforward and feedback at different levels of sensory hierarchy is at the heart of many predictive processing theories of hallucinations (Wilkinson, 2014; Sterzer et al., 2018). In general, examining the thalamus with high spatiotemporal resolution is within the reach of state-of-the-art BOLD fMRI (Figure 3), and could provide much needed insight into PSD etiology, stratification and treatment monitoring. These lines of investigation can be augmented by parallel work in the pharmacological domain, which we discuss next.

### Pharmacological neuroimaging

Studies have concluded that, although PSD has a strong heritable component, there is considerable heterogeneity in the types of genetic changes underlying this syndrome (Chumakov et al., 2002; Straub et al., 2002). This has made it difficult to look for mechanistic insights based on analysis at the genetic level due to small effects across many genes (Craddock et al., 2007). Luckily, a number of pharmacological manipulations are known to replicate behavioral features of PSD, and therefore offer a tool to uncover mechanisms. Cardinal examples are ketamine, lysergic acid diethylamide (LSD), psilocybin, and delta-9-tetrahydrocannabinol (THC) (Paparelli et al., 2011; Steeds et al., 2015). The fact that these compounds induce key symptoms of PSD, yet have fundamental differences in their molecular site of action, indicates that overemphasizing the molecular level of analysis in both explaining and treating PSD symptoms may be misplaced. Critically, some of the large-scale neural system perturbations that impinge upon associative thalamocortical processing appear to be impacted by these diverse compounds (Höflich et al., 2015; Avram et al., 2022). It is also worth repeating that PSD symptomatology is quite heterogeneous and similarly these various compounds may individually induce stereotypical behaviors, but collectively reproduce this heterogeneity.

Ketamine is a non-competitive NMDA receptor antagonist that rapidly and transiently induces symptoms seen in PSD when administered at subanesthetic doses, predominantly impacting executive function (Krystal et al., 2003). A working model is that ketamine at these doses (i.e., 0.6 mg/kg) preferentially blocks NMDA receptors on interneurons compared to excitatory neurons resulting in output disinhibition (i.e., causing an increase in excitatory/inhibitory E/I ratio). At the mesoscopic scale, this results in reduced precision of neural representation underlying short term memory and integration (Morgan and Curran, 2006). At the macroscale, these changes translate to large-scale network perturbations that underlie the various symptoms of PSD (Olney et al., 1999). In support of these ideas, one study (Höflich et al., 2015) showed that administration of ketamine leads to alterations in thalamic functional connectivity at rest similar to the patterns reported in PSD. Anticevic et al. (2012b) showed that ketamine administration in a spatial working memory task altered task-evoked activity patterns that were also accompanied by altered resting state functional connectivity in the same networks. This experimental observation was captured by a neural model that simulates ketamine’s effect by elevating E/I ratio (Anticevic et al., 2012b). Given that thalamic inputs have recently been shown to independently modulate E and I components of cortical networks (Mukherjee et al., 2021), future models that incorporate thalamic microcircuits will provide important insights into how changes in E/I balance across distributed cortical systems may contribute to the dysconnectivity patterns seen under ketamine, and ultimately in PSD itself (Mukherjee et al., 2020; Lam et al., 2022).

Lysergic acid diethylamide (LSD) is a psychedelic substance, which activates serotonin 5HT2A receptors and induces symptoms of PSD with a dominant effect on perception and thought organization (De Gregorio et al., 2016; Preller et al., 2019). As with ketamine, LSD administration alters connectivity across thalamocortical circuits (Preller et al., 2019). Specifically, LSD appears to enhance thalamic connectivity to a number of cortical areas at rest, which may be related to altered sensory experiences under LSD (Müller et al., 2017). It will be interesting to investigate the specific impact of LSD on associative thalamic structures and their engagement in reasoning operations. In fact, this particular avenue of research may be quite critical in testing cognitive explanations for perceptual disruption in PSD (Avram et al., 2022).

Tetrahydrocannabinol is a compound that activates endogenous endocannabinoid receptors, which are known to modulate E/I balance across cortical circuitry (den Boon et al., 2015). Administering THC in otherwise healthy humans elicits key symptoms of PSD, including delusional thought content (Kleinloog et al., 2014). Similar to both ketamine and LSD, THC also perturbs large-scale neural system organization, and reproduces thalamocortical dysconnectivity to some extent (Camchong et al., 2017; Cupo et al., 2021).

Collectively, these human neuroimaging studies highlight the value of using pharmacology as a tool to perturb the brain in a manner that reproduces core symptoms of PSD. A major conclusion of these studies is that thalamocortical dysconnectivity may be an obligatory intermediate phenotype when mimicking PSD behavior. If true, this supports an etiological role of these circuits in psychosis. Future studies can exploit the individual differences within and across these manipulations in order to make even more precise mechanistic discoveries at the neural systems level. It is worth noting that the speed of these interventions provides therapeutic hope. For example, the fact that, under some conditions, certain individuals can experience well-formed delusions indicates that this feature of PSD may not require years of aberrant neural processing in order to form (Favrat et al., 2005), but rather may be the result of a rapid perturbation in executive control of mnemonic processing (Krystal et al., 2000; Honey et al., 2004). This provisional conclusion indicates that perhaps reversing these symptoms may also be achievable with mechanistically-informed targeted approaches.

### Computational modeling

As described above, pharmacological neuroimaging can reveal how a molecular perturbation produces systems-level effects in brain-wide neural dynamics, which in turn correlate with evoked symptoms. Such experimental studies highlight the explanatory gaps across levels of analysis–from receptors to circuit, and behavior–that need to be bridged to have a mechanistic understanding of how disease processes and therapeutics impact system-level brain function and in turn relate to symptoms. One approach to integrate findings across levels is through computational modeling of neural systems. Computational models of thalamic and cortical circuits offer opportunities to study the impact of hypothesized molecular alterations on neural dynamics and cognitive function relevant to PSD and other neuropsychiatric disorders.

Recent studies using biophysically grounded circuit models have characterized mechanisms for how alterations to excitation-inhibition balance can impact neural function and cognitive behavior. For instance, in a thalamic circuit model inhibition via the reticular nucleus enables effective regulation of thalamic processing by top-down cortical control signals (Min, 2010). Biophysically grounded models of association cortical circuits have been developed for core cognitive functions such as decision making. In these models, perturbations of excitation-inhibition balance impacts the integration of evidence for decision making leading to behavioral deficits (Lam et al., 2022). An important future direction is to complement these models with performance optimized artificial neural networks that can also be fit to patient data. Such models can simultaneously do so while also making predictions about neural signals (Hummos et al., 2022). Another exciting direction is to combine modeling with theoretical analysis, which would answer “*why*” questions regarding thalamocortical organization (Wang and Halassa, 2022).

Computational models of large-scale brain network dynamics have also been productively applied to clinical and pharmacological neuroimaging. These models typically simulate a brain region in terms of its overall neural activity level (Murray et al., 2018; Shine et al., 2021). Correlated fluctuations across regions arise from inter-regional interactions via long-range projections which can be estimated in humans via diffusion tractography. Such models have been applied to simulate resting-state fMRI in patients with schizophrenia, and pharmacological effects of LSD. A recent focus in this literature underscores the importance of heterogeneity of local circuit properties across brain regions, such as hierarchical specialization from sensory to associative cortical regions (Demirtas et al.2019). Prior modeling studies have focused on cortico-cortical interactions but have yet to incorporate thalamic nuclei. Future modeling incorporating thalamo-cortical interactions can help explore the role of associative thalamus in shaping large-scale brain network dynamics and their alteration in PSD or by pharmacology.

## A translational framework for thalamocortical system targeting in PSD

Targeting the thalamus with deep brain stimulation (DBS), or more recently non-invasive ultrasound, has been useful in a number of clinical applications. Could a similar approach be useful in targeting treatment resistant PSD? This question is quite pertinent as ∼30% of patients continue to experience debilitating symptoms despite treatment with clozapine as their last resort antipsychotic agent (Lally et al., 2016).

As highlighted throughout this perspective, the thalamus occupies a central location not only in forebrain structural organization but its functional one as well. This conclusion is supported by both invasive recordings in non-human animals and neuroimaging studies in humans. The animal studies provide mechanistic insight that is hard to obtain from human studies, whereas the human studies provide direct relevance to cognitive operations that are difficult to implement in animals. Therefore, the combination of these approaches is necessary and jointly support the idea that associative thalamic structures are engaged in regulating the interactions within and across associative cortical areas, and that perturbations within these areas lead to a host of abnormalities characteristic of PSD.

This mesoscopic-to-macroscopic level of description is critical for bridging the findings at the molecular and cellular level with PSD symptoms, and ultimately develop the next generation diagnostics and therapeutics. Consistent with this notion, a recent circuit study on mice identified two distinct cell types in the mediodorsal thalamus that either enhance or suppress task-relevant prefrontal activity patterns (Mukherjee et al., 2021). The key bridging link to schizophrenia is that these cell types straddle two major hypotheses of its etiology at the molecular level–dopamine and parvalbumin interneurons. Specifically, the mediodorsal cell type that suppresses prefrontal activity patterns does so through the preferential innervation of prefrontal parvalbumin interneurons, and the experimental suppression of this thalamic subpopulation leads to a “jumping to conclusions” phenotype reminiscent of human PSD (Evans et al., 2015). This is because these thalamic cells preferentially suppress unreliable cortical inputs based on their computation for sensory uncertainty, allowing for appropriate deliberation when faced with noisy evidence. The second thalamic cell type is one that expresses the dopamine D2 receptor, and its activation of prefrontal disinhibitory interneurons (VIP+) enhances prefrontal inputs. This allows the prefrontal cortex to more effectively process sparse inputs by boosting these otherwise faint signals. Interestingly, a number of studies examining D2 binding in the human thalamus show that it is diminished in schizophrenia (Talvik et al., 2003; Yasuno et al., 2004). This is particularly salient in associative thalamic regions that form loops with the prefrontal cortex. Understanding how these changes relate to thalamic regulation of prefrontal dynamics may be a critical next step, and may clarify whether existing therapeutic strategies (e.g., D2 antagonists) are helpful or, in fact, add to the existing deficits. Given the noted PSD heterogeneity, one testable hypothesis is that certain PSD patients with thalamic D2 reduction are particularly impaired in appropriate prefrontal functions (e.g., working memory and task switching). This hypothesis can be tested through a combination of task-based fMRI and D2 PET imaging. In this context, resting state fMRI may serve as an additional tool of stratifying patients, and ultimately predicting their response to different types of treatments (including existing classes of antipsychotics). Such studies have the capacity to inform rational and patient-specific selection strategies for clinical trial design.

The studies above highlight an important idea; translational progress may be best achieved when it focuses on evolutionarily conserved areas between the human brain and its non-human counterparts. It is no secret that the human cortex is one of the most fascinating pieces of machinery nature has generated over the last 4 billion years of evolution on this planet. However, its unique expansion poses a challenge, insofar that many of its divisions are difficult to map on species normally studied in the laboratory (including macaque monkeys). The thalamus may provide unique translational opportunities in that domain; there are far fewer disputes about homology across divisions of the thalamus between species (even with mice) compared to, say, areas of frontal cortex (Neubert et al., 2014; Carlén, 2017).

That said, we should mention that the mechanistic accessibility that rodents provide needs to be augmented by the cognitive complexity that non-human primate models afford. These organisms, which can perform much higher level cognitive functions including well-parametrized versions of cognitive tasks used in PSD clinical practice (Castner et al., 2004; Ebitz et al., 2020), are indispensable in the larger context of determining the precise circuit perturbations in PSD.

Similar to non-human primates, our view is that computational models will play essential bridging roles across levels of analysis in the quest for next generation PSD diagnostics and therapeutics. In addition to biophysically constrained models, which provide a link from molecules to circuit function (Wang, 2010), recent advances in task-optimized neural network models promise to bridge circuit function to behavior (Wang et al., 2018; Richards et al., 2019; Schrimpf et al., 2020). The integration of these two modeling traditions, with the additional constraints offered by normative behavioral models (Collins and Shenhav, 2022), is likely going to be key in the community’s broader goal of understanding psychosis. In fact, there will be certain questions that may be impossible to test in animals (including humans), in which different classes of neural models end up being a better testbed (e.g., specific computational theories of cognition that require dynamical perturbations that cannot be experimentally implemented at this point). Importantly, effort in the direction of imposing brain-inspired architectural constraints on these task-optimized networks, suggest that we may even be able to use them to understand the specific role(s) of associative thalamic circuits in cognitive computations and how they can be leveraged to rescue cognitive abnormalities in PSD.

## Conclusion

The thalamus has emerged as a critical node in large-scale functional forebrain organization. Its continued exploration may offer mechanistic insights into how the brain implements cognitive operations whose perturbation, we have argued, form the basis of most PSD symptoms. Given the recent advances in targeting the thalamus through DBS and focused ultrasound, this new understanding may tell us where in the thalamus to target and when in the illness to do so.

## Data availability statement

The original contributions presented in this study are included in the article/supplementary material, further inquiries can be directed to the corresponding authors.

## Author contributions

AA and MH conceptualized and wrote the manuscript. Both authors contributed to the article and approved the submitted version.
